# Risk prediction of biomarkers for early multiple organ dysfunction in critically ill patients

**DOI:** 10.1186/s12873-021-00534-z

**Published:** 2021-11-08

**Authors:** Shigeto Ishikawa, Yuto Teshima, Hiroki Otsubo, Takashi Shimazui, Taka-aki Nakada, Osamu Takasu, Kenichi Matsuda, Junichi Sasaki, Masakazu Nabeta, Takeshi Moriguchi, Takayuki Shibusawa, Toshihiko Mayumi, Shigeto Oda

**Affiliations:** 1 Department of Emergency Medicine, University of Occupational and Environmental Health, 1-1 Iseigaoka, Yahata-nishi, Kitakyushu, 807-8555 Japan; 2 Department of Emergency and Critical Care Medicine, Chiba University Graduate School of Medicine, Chiba, Japan; 3 Department of Emergency and Critical Care Medicine, Kurume University School of Medicine, Kurume, Japan; 4 Department of Emergency and Critical Care Medicine, University of Yamanashi, Faculty of Medicine, Yamanashi, Japan; 5 Department of Emergency and Critical Care Medicine, Keio University School of Medicine, Tokyo, Japan

**Keywords:** Critically ill, Interleukin, Multiple organ dysfunction, Predictive marker, qSOFA

## Abstract

**Background:**

Shock and organ damage occur in critically ill patients in the emergency department because of biological responses to invasion, and cytokines play an important role in their development. It is important to predict early multiple organ dysfunction (MOD) because it is useful in predicting patient outcomes and selecting treatment strategies. This study examined the accuracy of biomarkers, including interleukin (IL)-6, in predicting early MOD in critically ill patients compared with that of quick sequential organ failure assessment (qSOFA).

**Methods:**

This was a multicenter observational sub-study. Five universities from 2016 to 2018. Data of adult patients with systemic inflammatory response syndrome who presented to the emergency department or were admitted to the intensive care unit were prospectively evaluated. qSOFA score and each biomarker (IL-6, IL-8, IL-10, tumor necrosis factor-α, C-reactive protein, and procalcitonin [PCT]) level were assessed on Days 0, 1, and 2. The primary outcome was set as MOD on Day 2, and the area under the curve (AUC) was analyzed to evaluate qSOFA scores and biomarker levels.

**Results:**

Of 199 patients, 38 were excluded and 161 were included. Patients with MOD on Day 2 had significantly higher qSOFA, SOFA, and Acute Physiology and Chronic Health Evaluation II scores and a trend toward worse prognosis, including mortality. The AUC for qSOFA score (Day 0) that predicted MOD (Day 2) was 0.728 (95% confidence interval [CI]: 0.651–0.794). IL-6 (Day 1) showed the highest AUC among all biomarkers (0.790 [95% CI: 0.711–852]). The combination of qSOFA (Day 0) and IL-6 (Day 1) showed improved prediction accuracy (0.842 [95% CI: 0.771–0.893]). The combination model using qSOFA (Day 1) and IL-6 (Day 1) also showed a higher AUC (0.868 [95% CI: 0.799–0.915]). The combination model of IL-8 and PCT also showed a significant improvement in AUC.

**Conclusions:**

The addition of IL-6, IL-8 and PCT to qSOFA scores improved the accuracy of early MOD prediction.

**Supplementary Information:**

The online version contains supplementary material available at 10.1186/s12873-021-00534-z.

## Background

Excessive immune response with the overproduction of inflammatory mediators leads to systemic inflammatory response syndrome (SIRS), which is crucial for the development of multiple organ dysfunction (MOD) [1]. MOD is an independent prognostic factor for intensive care unit (ICU) mortality [2]. Patients with MOD have longer ICU stays and higher mortality rates [3, 4]. The early detection of MODS may help identify patients at a risk of prolonged illness and death. Therefore, predicting early MOD can improve patient outcomes and quality of care.

Sequential organ failure assessment (SOFA) is a scale used to score organ failure and predict mortality by determining disease severity [5]. However, blood tests, including those of arterial blood gases, are essential to assess SOFA. In contrast, quick SOFA (qSOFA) was designed as a simple tool that can be used in the emergency department (ED) and can be used without performing blood tests [6]. qSOFA is considered a useful index for identifying patients with high mortality and those requiring systemic management in the ICU and is highly convenient in primary care [7]. In addition, the in-hospital mortality predictive effectiveness of qSOFA was statistically higher than that of SOFA in cases of suspected infection outside the ICU [8]. However, qSOFA did not outperform SOFA for patients in the ICU. qSOFA is affected by the severity of infection and quality of the health care system, and factors such as biomarkers that correlate with systemic inflammation are not included in the score. Therefore, the addition of simple blood tests, including those of cytokines, as in the present study, may improve the diagnostic accuracy of qSOFA for sepsis [9–11].

Interleukin (IL)-6 is a proinflammatory cytokine released by immune cells and reflects the degree of hypercytokinemia involved in systemic inflammatory changes [12]. IL-6 peaks at 6 h after invasion and is induced earlier than C-reactive protein (CRP) and procalcitonin (PCT). Furthermore, IL-6 enables earlier diagnoses of SIRS, and IL-6 levels reflect the severity and outcome of sepsis [13–15]. For these reasons, we focused on IL-6. We hypothesized that IL-6 could predict early MOD and that the addition of biomarkers to qSOFA could improve the prediction accuracy. The objective of this study is to investigate the early MOD prediction of biomarkers including IL-6 and the improvement of prediction accuracy by combining qSOFA with biomarkers.

## Methods

### Settings

This study was conducted using data from EDs and ICUs from 5 university hospitals (University of Occupational and Environmental Health, Chiba University, Kurume University, University of Yamanashi and Keio University) in Japan as a secondary analysis of a study [16]. The mother study aimed to identify the biomarker with the highest predictive value for late-phase MOD in critically ill patients. Various biomarkers were measured at three timepoints (days 0, 1, and 2). They evaluated predictive values for MOD (primary outcome, MOD on day 7 [late-phase]; secondary outcome, MOD on day 3 [early-phase]). This study was approved by the Institutional Review Board of the University of Occupational and Environmental Health (approval number H28–120).

### Study population

This study included patients with SIRS between September 2016 and September 2018. Patients were enrolled consecutively. The inclusion criteria were patient emergency admission (ICU or ED), age ≥ 20 years at the time of obtaining consent, patients predicted to hospitalized for at least 48 h by each physician in charge and patients with a diagnosis of SIRS according to the American College of Chest Physicians/Society of Critical Care Medicine criteria on admission [17]. In cases consent could not be obtained from the patient himself/herself due to impaired consciousness or dementia, consent was obtained from a spouse, relative, or other substitute. Patients with trauma were included if they had multiple injuries (≥2 injured area) and had a predicted Injury Severity Score ≥ 10, and patients with burns were included if they had a Burn Index ≥15. Patients who received steroids, immunosuppressive drugs, or preparations that affected serum IL-6 concentration within 1 week before emergency transport or ICU admission, those with HIV infection, those who were pregnant, or those considered ineligible for enrollment were excluded.

### Data collection

The patient information included demographic characteristics, etiology of admission, comorbidities, presence of hemodynamic instability, and history of treatment in the ED/ICU. Blood samples were collected 6 h after admission (Day 0) and the following morning (Day 1). Blood samples on Day 2 were collected during the morning period. We measured a suite of biomarkers to see which ones improved prediction. The inflammatory biological markers included CRP, IL-6, IL-8, IL-10, tumor necrosis factor (TNF)-α, and PCT. For biomarkers other than IL-6, we selected them that have been widely studied as markers of inflammation [18–22]. Serum CRP levels were measured immediately using commercially available assays at each hospital; IL, TNF-α, and PCT levels were measured at outside facilities after serum samples were frozen and stored at − 20 °C (IL-6 and PCT were measured at Roche diagnostics K.K., TNF-α, IL-8, IL-10 were measured at SRL,inc). The biomarkers were determined using reagent kit for IL-6 and PCT (Elecsys IL-6 and Elecsys BRAHMS PCT), Roche Diagnostics GmbH, Mannheim, Germany; IL-8 and IL-10 (BIOSOURCE IL-8 EASIA kit and BIOSOURCE IL-10 EASIA kit), BioSource Europe S.A., Nivelles, Belgium; TNF-α (Quantikine HS ELISA Human TNF-α Immunoassay), R&D Systmes, Inc., Minneapolis USA. MOD was defined as two or more organs with a SOFA score ≥ 2. qSOFA and SOFA scores were recorded on Days 0, 1, and 2. The Acute Physiology and Chronic Health Evaluation (APACHE) II scores were recorded on admission.

### Statistical analysis

The outcome as an early MOD was assessed on Day 2 because reversible derangements induced by the inciting event or incomplete resuscitation may be reflected 24 h after admission. To evaluate the accuracy of predicting MOD by adding biomarkers to qSOFA, we developed a baseline model to predict MOD (Day 2) using a logistic regression analysis. We calculated the area under the curve (AUC) by drawing an ROC curve for the blood concentration of each biomarker on Days 0 and 1 to determine which biomarker and time points were most predictive of MOD on Day 2. We did a subgroup analysis with groups such as ED/ICU and infection/trauma/burn. The biomarkers and days with the highest AUC were used in the model added to qSOFA to evaluate whether the model improves the accuracy of predicting MOD. ROC curves were drawn, and AUC values were compared between the baseline model and the model with the biomarker added to qSOFA. The calculation of the 95% CI of AUC and testing for differences in AUC were performed based on the method of DeLong [23]. We calculated the net reclassification improvement (NRI) to compare each model and presented the values with 95% confidence intervals (CIs) [24]. The results were compared using Mann-Whitney U tests and chi-square tests. Multiple testing correction was applied using Sidak’s correction. *P*-values < 0.05 were considered statistically significant. All statistical analyses were performed using R version 3.5.3 and JMP 13.2.1. This study was analyzed by an independent statistician.

## Results

**Fig. 1:**
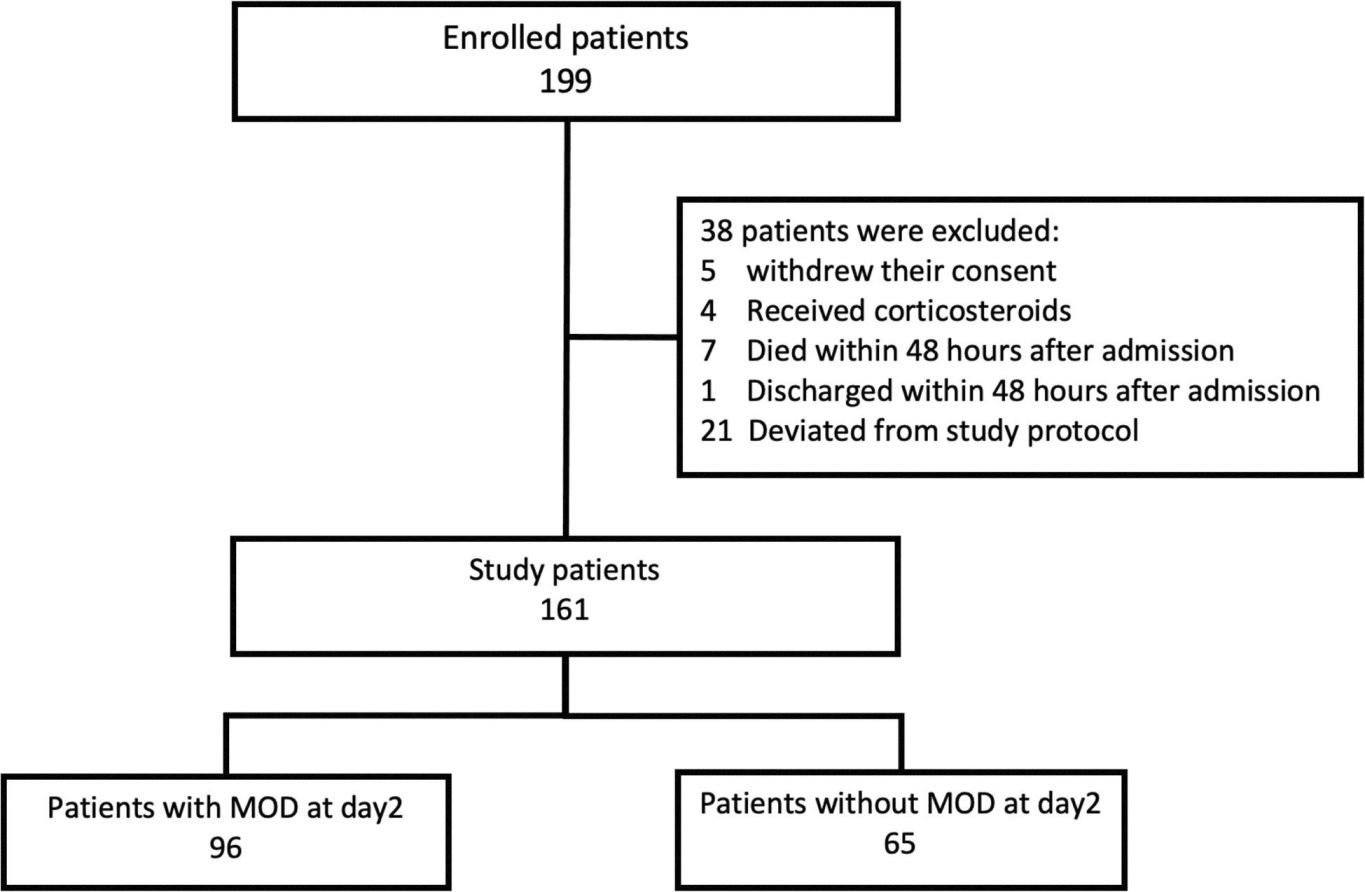
Patient selection flow diagram. A total of 199 patients were enrolled in the present study and screened for eligibility. Of those patients, 161 met the inclusion criteria. Overall, 96 (59.6%) patients experienced MOD on Day 2. MOD, multiple organ dysfunction

Of 199 patients enrolled in the study: 5 who withdrew their consent, 4 who were on steroids before admission, 7 who died within 48 h of admission, 1 who was discharged within 48 h of admission, and 21 who had deviated from the protocol for biomarker testing and SOFA score calculation due to missing samples or improper timing of the sampling. These 31 patients were excluded (Fig. 1). Of 161 eligible patients, 99 patients had MOD on Day 0. On Day 1, 9 patients recovered from MOD and 12 patients developed new MOD, for a total of 102 patients with MOD. On Day 2, 7 patients recovered from MOD and 1 patient had new MOD, for a total of 96 patients with MOD. No patients died by Day 2. Patients with MOD had significantly higher qSOFA, SOFA, APACHE II scores, mortality rates, fewer ICU-free days, ventilator-free days, renal replacement therapy (RRT)-free days (Table 1).

**Table 1:** Characteristics and clinical outcomes in patients with or without multiple organ dysfunction on Day2

	MOD (*n* = 96)	without MOD (*n* = 65)	***P*** value
**Characteristics**			
Age, years	70 (59–78)	72 (64–85)	0.145
Male sex, n(%)	59 (61.5)	42 (64.6)	0.684
Etiology of SIRS, (%)			
Infection	63 (65.6)	46 (70.8)	0.493
Post-surgery	8 (8.3)	4 (6.2)	0.605
Trauma	14 (14.6)	8 (12.3)	0.680
Burn	1 (1.0)	0 (0.0)	0.409
Acute pancreatitis	6 (6.3)	3 (4.6)	0.658
Others	10 (10.4)	6 (9.2)	0.805
APACHEII score on admission	29 (23–37)	19 (13–22)	< 0.001*
qSOFA on admission			< 0.001*
0	1	14	–
1	33	29	–
2	35	21	–
3	26	1	–
qSOFA≧2 on admission, n(%)	61 (64.2)	22 (33.8)	< 0.001*
SOFA score on admission			
Total SOFA score	10 (7–13)	3 (2–5)	< 0.001*
Respiration, (%)	72 (75.0)	23 (37.1)	< 0.001*
Coagulation, (%)	29 (30.2)	4 (6.2)	< 0.001*
Liver, (%)	14 (14.7)	4 (6.2)	0.092
Cardiovascular, (%)	58 (60.4)	3 (4.6)	< 0.001*
Central Nervous System, (%)	67 (69.8)	9 (13.8)	< 0.001*
Renal, (%)	46 (47.9)	9 (13.8)	< 0.001*
**Outcome**			
In hospital 28 day mortality	16 (16.7)	2 (3.1)	0.007*
ICU free days	13 (0–20)	26 (23–28)	< 0.001*
Ventilator free days	16 (1–24)	28 (28–28)	< 0.001*
RRT free days	25 (11–28)	28 (28–28)	< 0.001*

*MOD* Multiple organ dysfunction, *SIRS* Systemic inflammatory response syndrome, *APACHE* Acute Physiology and Chronic Health Evaluation, *qSOFA* Quick Sequential Organ Failure Assessment, *ICU* Intensive care unit, *RRT* Renal replacement therapy

Data are presented as median and interquartile range for continuous variables and *exact number (%) for categorical variables. P-*values were calculated using Pearson’s chi-square test or the Wilcoxon test. For APACHE II score, *n* = 93 with MOD and *n* = 37 without MOD

Each biomarker showed higher values in patients with MOD, except for WBC. CRP and PCT peaked after day 1, while IL-6, IL-8 and IL-10 peaked on Day 0 (Table 2). The ROC analysis of each biomarker on Day 0 and 1 was performed to assess MOD on Day 2. The results showed that IL-6 on Day 1 had the highest AUC (AUC: 0.790; 95% CI: 0.711–0.852), followed by IL-8 (AUC: 0.789; 95% CI: 0.712–0.850) (Table 3). On Day 0, PCT showed the highest AUC (AUC: 0.728; 95% CI: 0.644–0.799). Subgroup analysis was performed in the same way. In the ICU admission group, PCT on Day 0 and IL8 and IL6 on Day 1 showed high AUC (Additional file 1). In the group with infection, PCT showed the highest AUC on Day 0, IL8 on Day 1, followed by IL6 (Additional file 2). In the group without infection, IL6 on Day 1 showed the highest AUC (Additional file 3). In the group without ICU admission, ROC analysis could not be performed because there was only one case with MOD on Day 2.

**Table 2:** Blood levels of each biomarker in patients with or without multiple organ dysfunction on day 0,1,2

**Day-0**	**All (*n*** **= 161)**	**MOD (*n*** **= 99)**	**without MOD (*n*** **= 62)**
Interleukin-6, pg/ml, median (IQR)	371.7 (151.7–2471)	1111 (175.7–6204)	210.7 (87.34–554.18)
Procalcitonin, ng/ml, median (IQR)	2.19 (0.34–9.3)	5.18 (1.27–38.08)	0.43 (0.15–2.12)
C-reactive protein, mg/dl, median (IQR)	10 (1–21.3)	12 (2.47–18.55)	3.95 (0.28–13.78)
White blood cell, 10^3^/μL, median (IQR)	13.2 (6.8–18.7)	11.1 (4.75–18.55)	15.15 (11.63–19.2)
Interleukin −8, pg/ml, median (IQR)	85.3 (23.88–335)	193.5 (54.35–965.5)	36.65 (15.95–93.1)
Interleukin-10, pg/ml, median (IQR)	10 (3–45)	17 (5–75.25)	5 (2–16)
Tumor necrosis factor-α, pg/ml, median (IQR)	3.91 (1.68–8.94)	5.1 (2.47–12.8)	2.33 (1.21–4.11)
**Day-1**	**All (n = 161)**	**MOD (*n*** **= 102)**	**without MOD (*n*** **= 59)**
Interleukin-6, pg/ml, median (IQR)	150.7 (57.99–1096)	394.6 (92.83–2163.25)	62.8 (38.4–137.85)
Procalcitonin, ng/ml, median (IQR)	3.3 (0.96–19.75)	7.19 (1.83–34.82)	1.61 (0.49–5)
C-reactive protein, mg/dl, median (IQR)	12 (6.79–21.3)	14.75 (7.72–25.53)	9.9 (5.85–17.05)
White blood cell, 10^3^/μL, median (IQR)	11.9 (8.3–16)	11.8 (7.33–15.98)	11.9 (9.15–16.35)
Interleukin −8, pg/ml, median (IQR)	41.1 (13.2–132)	89.55 (24.8–299.8)	14.2 (9.4–44)
Interleukin-10, pg/ml, median (IQR)	4 (2–12.5)	7 (3–24)	2 (2–4)
Tumor necrosis factor-α, pg/ml, median (IQR)	3.34 (1.94–6.04)	4.04 (2.21–8.2)	2.67 (1.6–4.11)
**Day-2**	**All (*n*** **= 161)**	**MOD (*n*** **= 96)**	**without MOD (*n*** **= 65)**
Interleukin-6, pg/ml, median (IQR)	102.1 (42.37–262.5)	159.6 (88.86–492.98)	47.53 (24.75–88)
Procalcitonin, ng/ml, median (IQR)	2.62 (0.83–14.62)	6.53 (1.54–25.88)	0.95 (0.39–3.93)
C-reactive protein, mg/dl, median (IQR)	15.5 (9–21.5)	18.1 (10.83–24.08)	11.9 (7–17)
White blood cell, 10^3^/μL, median (IQR)	10.7 (8.2–14.2)	11.5 (7.38–14.75)	9.7 (8.3–13)
Interleukin −8, pg/ml, median (IQR)	25.3 (11.8–82.3)	55.75 (20.58–120.5)	11.8 (5.6–24.4)
Interleukin-10, pg/ml, median (IQR)	2 (2–7)	4 (2–9.5)	2 (2–2)
Tumor necrosis factor-α, pg/ml, median (IQR)	2.93 (2.02–4.75)	3.7 (2.35–5.42)	2.27 (1.77–3.17)

**Table 3:** Receiver operating characteristic curve analysis for prediction of multiple organ dysfunction on day 2 for various biomarkers on day 0,1

	AUC	95%CI
Day-0		
Interleukin-6	0.647	0.558–0.727
Procalcitonin	0.728	0.644–0.800
C-reactive protein	0.603	0.513–0.686
White blood cell	0.563	0.648–0.804
Interleukin −8	0.717	0.631–0.790
Interleukin-10	0.619	0.527–0.704
Tumor necrosis factor-α	0.633	0.540–0.717
Day-1		
Interleukin-6	0.790	0.711–0.852
Procalcitonin	0.705	0.618–0.780
C-reactive protein	0.610	0.519–0.693
White blood cell	0.457	0.687–0.835
Interleukin −8	0.789	0.711–0.850
Interleukin-10	0.751	0.669–0.817
Tumor necrosis factor-α	0.635	0.544–0.716

*AUC* Area under the curve, *CI* Confidence interval

**Fig. 2:**
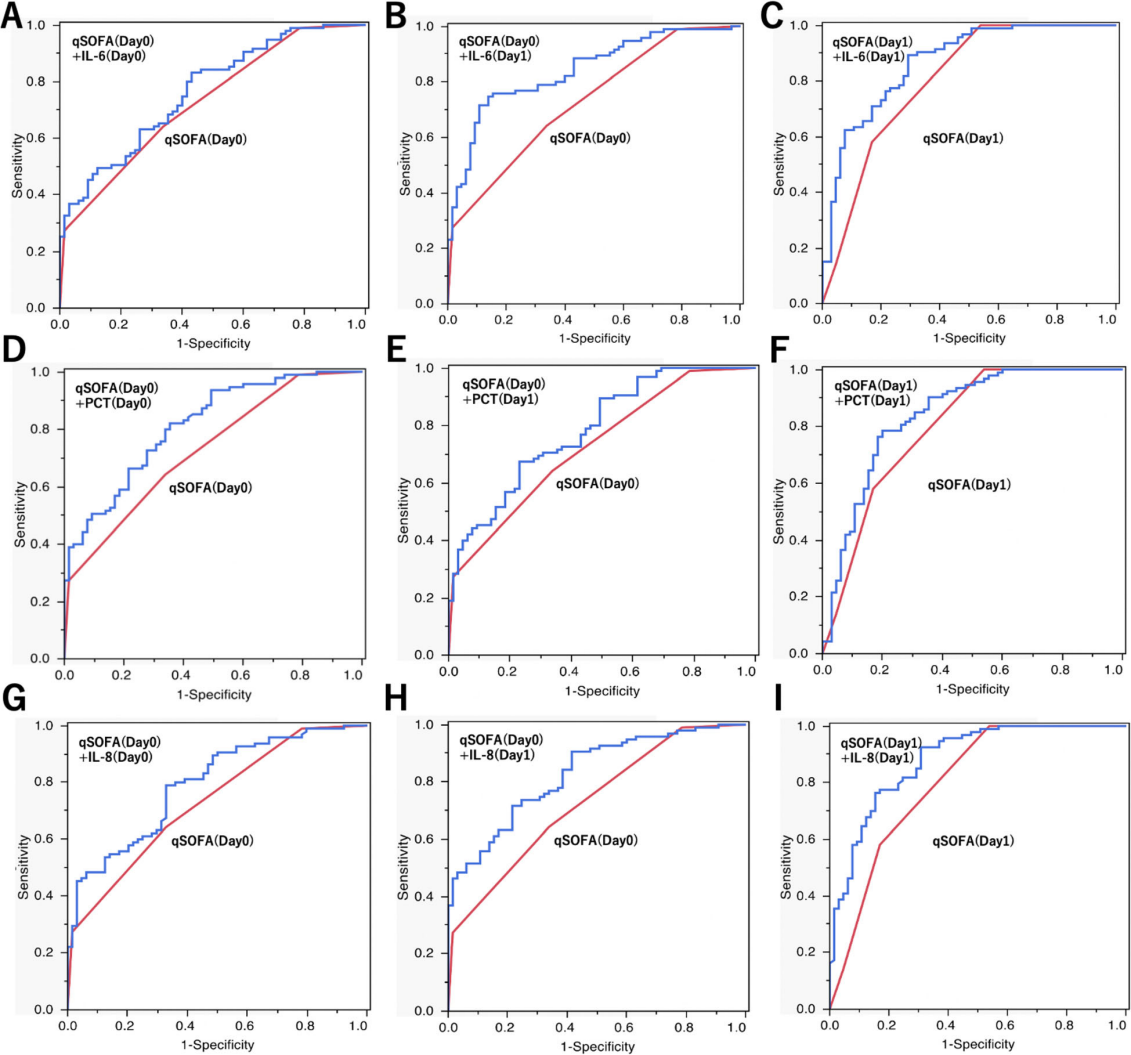
Prognostic value of a combined approach of IL-6, IL-8, PCT and baseline qSOFA**. A** AUC for baseline qSOFA (Day 0) with IL-6 (Day 0). **B** AUC for baseline qSOFA (Day 0) with IL-6 (Day 1). **C** AUC for baseline qSOFA (Day 1) with IL-6 (Day 1). **D** AUC for baseline qSOFA (Day 0) with PCT (Day 0). **E** AUC for baseline qSOFA (Day 0) with PCT (Day 1). **F** AUC for baseline qSOFA (Day 1) with PCT (Day 1). **G** AUC for baseline qSOFA (Day 0) with IL-8 (Day 0). **H** AUC for baseline qSOFA (Day 0) with IL-8 (Day 1). **I** AUC for baseline qSOFA (Day 1) with IL-8 (Day 1). Comparison of AUC revealed that the combination model using additional serum IL-6 concentration on Days 0 and 1 had a significantly higher AUC than the baseline model that uses only the qSOFA score. AUC = area under the curve, qSOFA = quick sequential organ failure assessment, IL = interleukin, PCT = procalcitonin

Next, as a baseline model, the predictive accuracy of MOD on Day 2 using Day 0 qSOFA scores was evaluated by logistic regression analysis (AUC: 0.728; 95% CI: 0.651–0.794). We developed a model combining qSOFA with IL-6, IL-8, and PCT, which showed high AUC. PCT was the biomarker that showed the highest AUC in the Day 0 combination (AUC: 0.814; 95% CI: 0.740–0.870; NRI: 0.661). The addition of Day 0 IL-6 significantly improved the NRI but did not significantly improve the AUC (AUC: 0.765; 95% CI: 0.685–0.830; NRI: 0.562). We analyzed the predictive accuracy of MOD (Day 2) in several models that individually added IL-6, IL-8, PCT (Day 1) to qSOFA (Day 0). In this additional model, IL-6 had the highest AUC compared to the baseline model (AUC: 0.842; 95% CI: 0.771–0.893; NRI: 0.802) (Table 4/Fig. 2). We also explored the possibility of further improving our accuracy by measuring qSOFA on Day 1. Changing qSOFA in this model from Day 0 to Day 1 and analyzing it in the same manner resulted in a significant improvement in AUC (AUC: 0.868; 95% CI: 0.799–0.915; NRI: 0.712). IL-8 and PCT also improved AUC predominantly in the same combination model.

**Table 4:** Predictive diagnostic accuracy of MOD on day 2 with qSOFA and additional Interleukin-6, Interleukin-8, Procalcitonin

	AUC(95%CI)	Improvement of AUC	*P*-value	NRI (95%CI)	*P*-value	Sensitivity	Specificity	PPV	NPV
qSOFA (Day-0)	0.728 (0.651–0.794)					64.2	66.2	73.5	55.8
qSOFA (Day-0) + IL-6(Day-0)	0.765 (0.685–0.830)	0.037 (0.001–0.073)	0.252	0.562 (0.267–0.857)	0.001*	83.2	56.9	73.8	69.8
qSOFA (Day-0) + IL-6(Day-1)	0.842 (0.771–0.893)	0.113 (0.053–0.174)	0.002*	0.802 (0.520–1.084)	< 0.001*	74.7	86.2	88.8	70.0
qSOFA (Day-0) + PCT (Day-0)	0.814 (0.740–0.870)	0.086 (0.033–0.138)	0.008*	0.661 (0.362–0.959)	< 0.001*	82.1	64.6	77.2	71.2
qSOFA (Day-0) + PCT (Day-1)	0.785 (0.707–0.847)	0.057 (0.011–0.103)	0.084	0.492 (0.188–0.797)	0.009*	67.4	76.9	81.0	61.7
qSOFA (Day-0) + IL-8(Day-0)	0.793 (0.716–0.854)	0.061 (0.016–0.105)	0.044*	0.502 (0.198–0.806)	0.007*	78.9	67.2	78.1	68.3
qSOFA (Day-0) + IL-8(Day-1)	0.829 (0.758–0.882)	0.100 (0.045–0.155)	0.002*	0.784 (0.494–1.073)	< 0.001*	71.6	78.5	82.9	65.4
qSOFA (Day-1)	0.801 (0.723–0.861)					58.1	83.1	83.1	58.1
qSOFA (Day-1) + IL-6(Day-1)	0.868 (0.799–0.915)	0.067 (0.025–0.108)	0.005*	0.712 (0.423–1.001)	< 0.001*	89.2	70.8	81.4	82.1
qSOFA (Day-1) + PCT (Day-1)	0.842 (0.765–0.897)	0.041 (0.009–0.073)	0.037*	0.503 (0.201–0.805)	0.003*	78.5	80.0	84.9	72.2
qSOFA (Day-1) + IL-8(Day-1)	0.881 (0.816–0.925)	0.080 (0.039–0.121)	< 0.001*	0.718 (0.423–1.013)	< 0.001*	92.5	69.2	81.1	86.5

*AUC* Area under the curve, *CI* Confidence interval, *IL* Interleukin, *NRI* Net reclassification improvement, *qSOFA* Quick squential organ failure assessment, *PPV* positive predictive value, *NPV* Negative predictive value

If the NRI is positive and the 95%CI does not straddle zero, the predictive ability of the new model is considered to be significantly improved from the baseline model

## Discussion

In this study, IL-6 on Day 1 was the most accurate biomarker for predicting early MOD. Furthermore, the addition of IL-6, IL-8, PCT blood levels to qSOFA was a more accurate predictor of early MOD. Although previous studies implicated that patients with MOD had poor prognosis, including increased mortality and hospitalization days [2, 3], this study also confirmed that patients with MOD on Day 2 had poor prognosis (Table 1).

Cytokine storms play an important role in the pathogenesis of MOD, and various cytokines have been assessed for their accuracy in predicting MOD. IL-6 is a cytokine involved in inflammatory responses and is released in response to tissue injuries and inflammatory stimuli, resulting in a physiological response. IL-6 acts locally and systemically and is a proinflammatory mediator and an anti-inflammatory regulator that stimulates anti-inflammatory cytokines, including IL-10 [25]. The mediators of acute inflammation and infection, such as CRP, IL-6, and PCT, have long been involved in the pathophysiology of critically ill patients and are routinely used for diagnostic, prognostic, and treatment monitoring in the ICU [25–27]. Compared with other biomarkers such as CRP and PCT, IL-6 responds more quickly to inflammatory stimuli, peaking at 3–6 h. This suggests that it has an advantage in predicting risk at admission. In this regard, several reports have shown that IL-6 is a useful biomarker for detecting early sepsis; however, CRP and PCT are still often within the reference range owing to their much slower rates [28–30]. On the other hand, some reports suggest that PCT is the predominant marker for the diagnosis of sepsis [31–33]. The AUC in Table 2 revealed that the predictive ability of IL-6 peaked on Day 1, and PCT had a higher AUC than IL-6 on Day 0. The subgroup analysis also showed that the prediction accuracy of PCT was significantly improved in the group with infection. This may indicate that IL-6 levels have an early increase in the blood but remain more reflective of MOD than other biomarkers after Day 1. In a study examining various cytokine concentrations in patients with severe sepsis, IL-6 and IL-8 in the first 24 h predicted organ dysfunction on Day 3 [18]. In light of these findings, the present study shows that IL-6 may predict early MOD with greater accuracy than other biomarkers.

However, it is cumbersome to accurately predict organ failure and mortality using a single biomarker alone; therefore, a combination of biomarkers and severity scores has been proposed to provide better results. Oberholzer et al. suggested that IL-6 and APACHE II scores were correlated and showed that these combined models increased the accuracy of predicting mortality in patients with severe sepsis [34]. In addition, a study examining factors that predicted mortality 90 days after ICU admission found that combining SAPS II with IL-6 and soluble suppression of tumorigenesis-2 improved prediction accuracy [35].

The accuracy of predicting post-hospital organ failure and in-hospital mortality using qSOFA has been previously reported [36, 37]. In contrast, a previous study reported that positive qSOFA scores had high specificity but poor sensitivity for predicting in-hospital mortality, acute organ dysfunction, and ICU admission in patients with infection outside the ICU, and the limits of qSOFA accuracy of the predictions have been reported [38]. The model individually added IL-6, IL-8, and PCT to qSOFA improved the accuracy of predicting early MOD in this study. Although CRP is a biomarker that is routinely used in clinical practice, it was excluded from the combination model with qSOFA because of its low AUC. Since qSOFA does not require testing, the improvement in predictive accuracy obtained without increasing healthcare costs is a major advantage. The combination in this study was qSOFA on admission and IL-6 on Day 1, which is also in line with that used in clinical practice. If qSOFA is positive on admission, it can be worthwhile to perform additional tests for IL-6,8 and PCT to predict early MOD. We also modeled qSOFA on Day 1 because even if qSOFA is negative on admission, it may be screened again on the next day. However, these results may be due to the fact that it is closer to Day2 when the MOD outcome is measured.

In the future, a new scoring system that combines severity scores and biomarkers to predict MODs needs to be investigated.

### Limitations

This study had some limitations. First, the sample size was small partly due to the difficulty in obtaining consent. This study included patients with a variety of diseases. Second, one of the problems with qSOFA is that it is difficult to assess consciousness in patients with cognitive impairment before the onset of infection [39]. In addition, the blood samples after Day 1 were collected during morning period. Therefore, the timing of blood collection on Day 0 could lead to some differences.

## Conclusion

We compared each biomarker as a predictor of MOD on Day 2. IL-6 on Day 1 had the highest predictive value. Furthermore, the adding IL-6, IL-8 and PCT, which had high AUC, to qSOFA score predicted MOD on Day 2 with greater accuracy. The present study showed that adding IL-6, IL-8 and PCT to qSOFA could predict early MOD.

## Supplementary Information

**Additional file 1.** Receiver operating characteristic curve analysis for prediction of multiple organ dysfunction on day 2 for various biomarkers on day 0,1 in the ICU admission group.

**Additional file 2.** Receiver operating characteristic curve analysis for prediction of multiple organ dysfunction on day 2 for various biomarkers on day 0,1 in the group with infection.

**Additional file 3.** Receiver operating characteristic curve analysis for prediction of multiple organ dysfunction on day 2 for various biomarkers on day 0,1 in the group without infection.

## Data Availability

The data of this study are available from the authors, but restrictions apply to the availability of these data, which are not publicly available. However, data are available from the authors upon reasonable request.

## Abbreviations

AUC: Area under the curve
qSOFA: Quick sequential organ failure assessment
IL: Interleukin
CRP: C-reactive protein
PCT: Procalcitonin
TNF: Tumor necrosis factor
sST2: Solublesuppressionoftumorigenesis-2
MOD: Multiple organ dysfunction
NRI: Net reclassification improvement
SIRS: Systemic inflammatory response syndrome
APACHE II: Acute Physiology and Chronic Health Evaluation II
ICU: Intensive care unit
