# Supplementary material for: Risk prediction of biomarkers for early multiple organ dysfunction in critically ill patients
Source: BMC Emerg Med. 2021 Nov 8;21:132. doi: 10.1186/s12873-021-00534-z (PMC8573766; doi:10.1186/s12873-021-00534-z)
Supplement: Supplementary file 1 — Additional file 1. Receiver operating characteristic curve analysis for prediction of multiple organ dysfunction on day 2 for various biomarkers on day 0,1 in the ICU admission group. [file 12873_2021_534_MOESM1_ESM.docx]

**Supplementary material 1. Receiver operating characteristic curve analysis for prediction of multiple organ dysfunction on day 2 for various biomarkers on day 0,1 in the ICU admission group.**

|  | **AUC** | **95%CI** | | **N** |
| --- | --- | --- | --- | --- |
| Day-0 |  |  |  | |
| Interleukin-6 | 0.684 | 0.570-0.773 | 131 | |
| Procalcitonin | 0.713 | 0.607-0.799 | 131 | |
| C-reactive protein | 0.573 | 0.464-0.676 | 131 | |
| White blood cell | 0.583 | 0.481-0.679 | 131 | |
| Interleukin -8 | 0.731 | 0.632-0.812 | 131 | |
| Interleukin-10 | 0.646 | 0.533-0.745 | 128 | |
| Tumor necrosis factor-α | 0.700 | 0.585-0.794 | 129 | |
| Day-1 |  |  | 131 | |
| Interleukin-6 | 0.787 | 0.693-0.859 | 131 | |
| Procalcitonin | 0.732 | 0.619-0.821 | 131 | |
| C-reactive protein | 0.578 | 0.468-0.682 | 131 | |
| White blood cell | 0.489 | 0.388-0.590 | 131 | |
| Interleukin -8 | 0.808 | 0.718-0.874 | 131 | |
| Interleukin-10 | 0.770 | 0.671-0.846 | 130 | |
| Tumor necrosis factor-α | 0.730 | 0.622-0.816 | 129 | |

AUC, area under the curve; CI, confidence interval
