# Supplementary material for: Risk prediction of biomarkers for early multiple organ dysfunction in critically ill patients
Source: BMC Emerg Med. 2021 Nov 8;21:132. doi: 10.1186/s12873-021-00534-z (PMC8573766; doi:10.1186/s12873-021-00534-z)
Supplement: Supplementary file 2 — Additional file 2. Receiver operating characteristic curve analysis for prediction of multiple organ dysfunction on day 2 for various biomarkers on day 0,1 in the group with infection. [file 12873_2021_534_MOESM2_ESM.docx]

**Supplementary material 2. Receiver operating characteristic curve analysis for prediction of multiple organ dysfunction on day 2 for various biomarkers on day 0,1 in the group with infection.**

|  | **AUC** | **95%CI** | | **N** |
| --- | --- | --- | --- | --- |
| Day-0 |  |  |  | |
| Interleukin-6 | 0.694 | 0.587 - 0.783 | 110 | |
| Procalcitonin | 0.825 | 0.732 - 0.891 | 110 | |
| C-reactive protein | 0.695 | 0.587 - 0.785 | 110 | |
| White blood cell | 0.581 | 0.472 - 0.683 | 110 | |
| Interleukin -8 | 0.778 | 0.677 - 0.854 | 109 | |
| Interleukin-10 | 0.665 | 0.555 - 0.760 | 107 | |
| Tumor necrosis factor-α | 0.678 | 0.564 - 0.775 | 107 | |
| Day-1 |  |  |  | |
| Interleukin-6 | 0.816 | 0.723 - 0.884 | 110 | |
| Procalcitonin | 0.765 | 0.661 - 0.844 | 110 | |
| C-reactive protein | 0.655 | 0.546 - 0.749 | 110 | |
| White blood cell | 0.437 | 0.334 - 0.545 | 110 | |
| Interleukin -8 | 0.874 | 0.792 - 0.927 | 110 | |
| Interleukin-10 | 0.778 | 0.681 - 0.852 | 110 | |
| Tumor necrosis factor-α | 0.710 | 0.605 - 0.797 | 109 | |

AUC, area under the curve; CI, confidence interval
