# Supplementary material for: Risk prediction of biomarkers for early multiple organ dysfunction in critically ill patients
Source: BMC Emerg Med. 2021 Nov 8;21:132. doi: 10.1186/s12873-021-00534-z (PMC8573766; doi:10.1186/s12873-021-00534-z)
Supplement: Supplementary file 3 — Additional file 3. Receiver operating characteristic curve analysis for prediction of multiple organ dysfunction on day 2 for various biomarkers on day 0,1 in the group without infection. [file 12873_2021_534_MOESM3_ESM.docx]

**Supplementary material 3. Receiver operating characteristic curve analysis for prediction of multiple organ dysfunction on day 2 for various biomarkers on day 0,1 in the group without infection.**

|  | **AUC** | **95%CI** | | **N** |
| --- | --- | --- | --- | --- |
| Day-0 |  |  |  | |
| Interleukin-6 | 0.606 | 0.440 - 0.750 | 49 | |
| Procalcitonin | 0.672 | 0.503 - 0.806 | 49 | |
| C-reactive protein | 0.515 | 0.348 - 0.680 | 49 | |
| White blood cell | 0.541 | 0.380 - 0.695 | 49 | |
| Interleukin -8 | 0.643 | 0.476 - 0.782 | 49 | |
| Interleukin-10 | 0.460 | 0.291 - 0.639 | 49 | |
| Tumor necrosis factor-α | 0.644 | 0.470 - 0.787 | 49 | |
| Day-1 |  |  |  | |
| Interleukin-6 | 0.785 | 0.618 - 0.892 | 49 | |
| Procalcitonin | 0.706 | 0.534 - 0.835 | 49 | |
| C-reactive protein | 0.605 | 0.431 - 0.756 | 49 | |
| White blood cell | 0.513 | 0.353 - 0.670 | 49 | |
| Interleukin -8 | 0.650 | 0.456 - 0.804 | 49 | |
| Interleukin-10 | 0.720 | 0.555 - 0.842 | 49 | |
| Tumor necrosis factor-α | 0.625 | 0.442 - 0.778 | 48 | |

AUC, area under the curve; CI, confidence interval
